# Rheumatic heart disease in Uganda: the association between MHC class II HLA DR alleles and disease: a case control study

**DOI:** 10.1186/1471-2261-14-28

**Published:** 2014-02-28

**Authors:** Emmy Okello, Andrea Beaton, Charles K Mondo, Paul Kruszka, Noah Kiwanuka, Richard Odoi-Adome, Juergen Freers

**Affiliations:** 1 Department of Medicine, Makerere University/Uganda Heart Institute, Ward 1C, Mulago Hospital Complex., PO Box 7051, Kampala, Uganda; 2 Uganda Heart Institute, Mulago Hospital Complex, Kampala, Uganda; 3 Department of Cardiology, Children’s National Medical Center, Washington, DC, USA; 4 National Human Genome Research Institute/NIH, Bethesda, USA; 5 Department of Epidemiology and Biostatistics, School of Public Health, Makerere University, Kampala, Uganda; 6 School of Pharmacy, Makerere University, Kampala, Uganda

**Keywords:** Rheumatic heart disease, HLA-DR1, HLA-DRB11, MHC- major histocompatibility complex

## Abstract

**Background:**

Rheumatic heart disease (RHD), the only long term consequence of acute rheumatic fever, remains a leading cause of morbidity and mortality among young adults in Uganda. An inherited susceptibility to acute rheumatic fever centers around the major histocompatibility class II human leucocyte antigens. However, there is paucity of data from sub-Saharan Africa. This study compares the frequency of HLA class II DR alleles between RHD cases and normal controls in Uganda.

**Methods:**

One hundred ninety-nine participants including 96 established RHD cases aged 5–60 years and 103 age and sex matched normal controls were recruited for participation. DNA was manually extracted from buffy coat samples and HLA analysis was performed. HLA-DR allelic frequency comparison between cases and controls were estimated using conditional logistic regression with 95% confidence intervals. P -values were corrected for multiple hypothesis testing.

**Results:**

199 participants (103 female, 51.8%) completed the study. The mean (SD) age in years for cases and controls were 29.6 (10.2) and 29(18), respectively. After conditional logistic regression and multiple hypothesis testing, HLA-DR1was associated with a decreased risk of RHD (OR = 0.42, CI 0.21-085, P = 0.01, Corrected P value (P_C_) = 0.09,) while HLA-DR11 was associated with increased risk of RHD (OR = 3.31, CI 1.57-6.97, P = <0.001, Pc < 0.001). No other significant associations were found.

**Conclusion:**

In this first study of HLA genetic susceptibility to RHD in Uganda, HLA- DR1 was more common in normal controls while HLA- DR11 was more common among RHD cases suggesting a disease susceptibility association. In future studies, high resolution HLA analysis and genome wide studies should be carried out to confirm this pattern.

## Background

Rheumatic heart disease (RHD) is currently thought to affect around 70 million people worldwide, with 1.4 million annual deaths [1]. The vast majority of these cases originate from central Asia and sub-Saharan Africa [2,3]. In Uganda, RHD is the most common cause of heart disease in patients between the ages of 15 and 49 [4].

Central to the complex pathogenesis of RHD is an inappropriate immune response triggered by an infection with rheumatogenic strains of Group A beta-haemolytic streptococcus. However, only 3-6% of those infected develop acute rheumatic fever [5], and genetic host susceptibility factors are thought to play a key role in disease development [6,7].

While there are many remaining questions, host susceptibility appears to center around the major histocompatibility complex (MHC) human leukocyte antigens (HLA) and other cellular antigen variants [8,9]. The MHC is a critical component of antigen presentation. Variations between HLA antigens can affect T-cell response and the resulting levels of cytokine production. It is these cytokines that play a role in the initial and ongoing cardiac damage seen in RHD. A number of MHC class II alleles including HLA-DR, DQ and DB loci have been implicated in this process [10,11]; however, a great deal of variability between patients from different ethnic backgrounds exists [11,12]. In Uganda, as in much of sub-Saharan Africa, there is a lack of data on genetic susceptibility to RHD. This is the first study to analyze HLA in RHD patients in Uganda and Eastern sub-Saharan Africa, and to our knowledge, the largest HLA study conducted.

## Methods

### Ethical approval

Ethical approval for the study was provided by the Institutional Review Board of the School of Medicine, Makerere University College of Heath Sciences and permission confirmed through the Uganda National Council for Science and Technology (UNCST).

The study conformed to the principles outlined in the declaration of Helsinki. Each study participant aged 18 years and above gave a written informed consent. Parents or next of kin gave written informed consent on behalf of the minor participants.

### Study design

A matched case control study was conducted comparing the frequency of MHC class II HLA- DR alleles between patients with established RHD and normal controls in a population of ethnic black Ugandans.

A total of 199 participants took part in the study. Ninety six established RHD cases, aged 5 to 60 years were recruited from the general cardiac outpatient clinic, pediatric cardiac clinic, and the general inpatient cardiology ward at the Uganda Heart Institute (UHI), Mulago Hospital Complex. All study participants underwent echocardiography and met criteria for definite RHD based on the 2012 World Heart Federation (WHF) guidelines for echocardiographic diagnosis of RHD [13]. One hundred and three controls were recruited from healthy relatives of patients being seen at the hospital for reasons other than RHD, and were age matched within five years of cases. All control subjects also underwent echocardiography to confirm that they did not have RHD. Other exclusion criteria included history or evidence of connective tissue disease, any abnormality noted on echocardiogram, and unwillingness to sign informed consent/assent.

For our power calculation, we used an anticipated difference of 10% between HLA frequency between cases and controls. In order to achieve an 80% chance of detecting an association between HLA and RHD (type I error rate of 5%), 88 cases and 88 controls would need to be recruited. The estimated sample size was increased to 100 per group to account for any losses.

### Study procedure

Following a written informed consent/assent, each study participant underwent a clinical examination followed by echocardiography according to the European society of cardiology guidelines [14].

### Sample preparation and DNA extraction

Six (6) milliliters of venous blood were obtained from each participant. Samples were centrifuged and buffy coat separated and stored at -80 degrees Celsius. During DNA extraction, the frozen sample was thawed and DNA extraction was preformed manually using the Genotype DNA isolation kit (Hain lifesciences, Nehren, Germany). DNA extraction and HLA analysis was conducted at the MBN DNA Laboratory, Kampala, Uganda.

### HLA-DR DNA polymerase chain reaction (PCR)

DNA typing for HLA-DR was performed through polymerase chain reaction (PCR) amplification (GeneAmp PCR System 9700, Applied Biosystems, California, USA) with Olerrup sequence specific primers (low resolution Olerup SSP® HLA Typing Kits, Stockholm, Sweden) according to protocol, as previously described [15]. The final amplicons were stored at 4 degrees Celsius until electrophoresis was performed.

Sequence-specific primers (PCR-SSP) distinguish specific alleles or groups of alleles by sequence differences at the 3' end of the primer [16]. After PCR amplification, the amplified DNA fragments were size-separated in 2.0% Agarose gel (PEQLAB Biotechnologies, Germany) stained with ethidium bromide and then visualized under UV light. The 100 bp DNA ladder (Solis BioDyne, Estonia**)** was used as the size marker.

### Statistical analysis

#### Statistical analysis

Allelic frequencies of HLA-DR from study participants were evaluated in STATA (Version 13, College Station, TX: Stata Corporation). The significance of the differences of between HLA-DR alleles was determined using Chi square analysis. Fisher’s exact test was used for small expected frequencies. P-values were considered statistically significant if its value was < 0.05. Conditional logistic regression analysis was conducted to determine odds ratios of the strength of association between HLA-DR alleles and RHD. P-values were further adjusted for multiple hypothesis testing by multiplying the P-values by the group number (9). Data was managed with Epi Data 3.0 (Odensk, Denmark) [17].

## Results

Demographic information for the 199 participants (96 cases and 103 controls) can be found in Table 1. Matching was successful with the ratio of male to female participants in each group being 1:1. The mean (SD) age in years for cases and controls were 29.6 (10.2) and 29 (18), respectively.

**Table 1:** Baseline characteristics

**Variable**	**Total**	**Cases**	**Controls**	**P value**
**Age**				
Mean (SD)	29.4(12.1)	29.6(10.2)	29.8(9.8)	0.88
**Sex**				
Male (N,%)	96(46.7%)	50(52%)	51(49.5%)	0.72

SD- Standard deviation.

Table 2 displays the results of HLA-DR allelic frequencies between the two groups. There was a lower frequency of HLA-DR1 in cases compared to controls, (14.6% versus 31.3%), and a higher frequency of HLA- DR11 in cases compared to controls (31.3% versus 10.7%). After conditional regression analysis and adjusting for multiple hypothesis testing, HLA-DR1 (OR = 0.42, CI 0.21-0.85, P = 0.01, Corrected P value (Pc) =0.09) was demonstrated to be associated with a decreased risk of RHD while HLA-DR11 (OR = 3.31, CI 1.97- 6.97, P < 0.0007, Pc = 0.006) was significantly associated with RHD (Table 3).

**Table 2:** HLA DR allelic frequencies between RHD cases and non RHD controls

**Variable**		**Total (n,%)**	**Cases (n,%)**	**Controls (n,%)**	**P value**
**HLA**	**DR1**	45/199, 22.6%	14/96, 14.6%	31/103, 30.1%	0.01
	**DR3**	18/199,9.04%	7/96, 7.29%	11/103, 10.7%	0.4
	**DR7**	16/199, 8.04%	11/96, 11.5%	5/103, 4.9%	0.1
	**DR8**	11/199, 5.53%	4/96, 4.2%	7/103, 6.8%	0.34
	**DR9**	7/199, 3.52%	4/96, 4.2%	3/103, 2.9%	0.36
	**DR10**	8/199, 4.02%	6/96, 6.3%	2/103, 1.9%	0.13
	**DR11**	41/199,20.60%	30/96, 31.3%	11/103, 10.7%	<0.001
	**DR13**	11/199, 5.53%	4/96, 4.2%	7/103, 6.8%	0.47
	**DR17**	24/199,12.06%	9/96, 9.4%	15/103, 14.6%	0.28

HLA- Human leukocyte antigen.

**Table 3:** Conditional regression analysis of association between HLA DR alleles and RHD

**Variable**	**OR (95% CI)**	**P value**	**Pc**
**HLA DR1**	0.42(0.21-0.85)	0.01	0.09
**HLA DR3**	0.65 (0.24-1.76)	0.4	
**HLA DR7**	2.5 (0.83-7.5)	0.1	
**HLA DR8**	0.54 (0.15-1.93)	0.34	
**HLA DR9**	2.08(0.42-10.3)	0.36	
**HLA DR10**	3.42 (0.68-17.27)	0.13	
**HLA DR11**	3.31(1.57-6.97)	0.0007	<0.006
**HLA DR13**	0.63(0.17-2.21)	0.47	
**HLA DR17**	0.62(0.26-1.47)	0.28	

Pc- Corrected P value for multiple hypothesis testing.

## Discussion

This is the first study of genetic susceptibility to RHD in Uganda. The results suggest that different class II MHC genes may result in both increased susceptibility to and increased protection from RHD. We found a significant association of HLA-DR11 in patients affected by RHD and a decreased risk of RHD in association with HLA-DR1.

In other populations, HLA-DR1 has been implicated as being both a protective factor and a susceptibility factor. Gundogdu et al., found a significantly higher frequency of HLA-DRB1*01 in controls compared to RHD patients [18]. However, Maharaj et al., found that patients with RHD had a higher frequency of HLA-DR1 compared to healthy controls [19]. Mixed results have also been reported for the HLA-DRB1*11 allele. Chou et al., showed an increase in HLA-DRB1*11 in RHD patients in Taiwan [7] but Haydardedeoglu et al. [20], and Guadalupe et al., [9], found HLA-DRB1*11 to be decreased in RHD patients from Turkey and Mexico, respectively. These differences in RHD associations across different populations support the concept of ethnic specific genetic susceptibility for HLA alleles [5]. Other explanations for the variability of studies include small sample sizes which may not capture rare HLA alleles, different typing methods (serologic versus DNA based), and heterogeneity of the causative agent. More than fifteen streptococcal strains have been associated with rheumatic fever and each distinct strain may select for a unique DR antigen, resulting in different susceptibility alleles [11].

Africa has one of the most genetically diverse populations [21], thus differences in HLA alleles is expected between various geographic locations in sub-Saharan Africa. In a study that examined HLA susceptibility in black South Africans, Maharaj et al., found an increase in HLA-DR6 in patients with RHD and no significant change in HLA-DR2 [19], while these alleles were absent in both cases and controls of our study. Carlquist et al., in a meta-analysis of HLA-DR association with RHD, showed that DR 1 was the susceptibility gene in blacks (South Africans and African Americans) and DR 3 and DR4 was the susceptibility genes in East Indians and American whites, respectively [11]. In our study, DR1 was not associated with RHD while DR3 was only marginally present (7 cases and 11 controls), and DR4 was absent.

A potential weakness of many HLA association studies, including ours, is a low level HLA typing which does not account for the multitude of polymorphisms found in the MHC [19]. Despite this weakness, ours is the only HLA study in RHD to have been conducted in Uganda [22] and the largest one [19,23,24].

A strength of this study is the homogeneity of the cases and controls as ethnic diversity and population stratification can weaken the association of HLA to RHD and cause spurious allelic associations [25]. The Ugandan population is mostly all black African with a small minority of Indians. In the present study all study participants were ethnic blacks.

More work needs to be done in genetic susceptibility to RHD and many questions remain unanswered. The HLA system is likely not the only important part of genetic susceptibility, and an unbiased search of the genome with a genome wide association study is warranted to give the full spectrum of genetic susceptibility to RHD.

## Conclusion

In this first study of the association between the MHC class II HLA-DR antigens and RHD in Uganda, we have found that HLA-DR1 was more common in healthy controls while HLA DR11 was more common among RHD cases suggesting a disease susceptibility association. Further studies, including high resolution HLA analysis and whole genome interrogations should be carried out to confirm this pattern.

## Competing interests

The authors declare that they have no competing interest.

## Authors’ contribution

EO, CM designed the study, EO, PK and AB did the molecular analysis, EO, PK and NK did the statistical analysis. EO, PK, NK, CM, AB, AB, CM, RO, JF wrote the manuscript. All authors read and approved the final manuscript.

## Pre-publication history

The pre-publication history for this paper can be accessed here:

http://www.biomedcentral.com/1471-2261/14/28/prepub
